# A Method for Quantification of Epithelium Colonization Capacity by Pathogenic Bacteria

**DOI:** 10.3389/fcimb.2018.00016

**Published:** 2018-02-01

**Authors:** Rune M. Pedersen, Rasmus B. Grønnemose, Kristian Stærk, Cecilie A. Asferg, Thea B. Andersen, Hans J. Kolmos, Jakob Møller-Jensen, Thomas E. Andersen

**Affiliations:** ^1^Research Unit of Clinical Microbiology, Department of Clinical Research, University of Southern Denmark, Odense University Hospital, Odense, Denmark; ^2^Department of Biochemistry and Molecular Biology, University of Southern Denmark, Odense, Denmark

**Keywords:** epithelial colonization, flow chamber, time-lapse fluorescence microscopy, host-pathogen interaction, uropathogenic *Escherichia coli*, shiga toxin-producing *Escherichia coli*, *Staphylococcus aureus*

## Abstract

Most bacterial infections initiate at the mucosal epithelium lining the gastrointestinal, respiratory, and urogenital tracts. At these sites, bacterial pathogens must adhere and increase in numbers to effectively breach the outer barrier and invade the host. If the bacterium succeeds in reaching the bloodstream, effective dissemination again requires that bacteria in the blood, reestablish contact to distant endothelium sites and form secondary site foci. The infectious potential of bacteria is therefore closely linked to their ability to adhere to, colonize, and invade epithelial and endothelial surfaces. Measurement of bacterial adhesion to epithelial cells is therefore standard procedure in studies of bacterial virulence. Traditionally, such measurements have been conducted with microtiter plate cell cultures to which bacteria are added, followed by washing procedures and final quantification of retained bacteria by agar plating. This approach is fast and straightforward, but yields only a rough estimate of the adhesive properties of the bacteria upon contact, and little information on the ability of the bacterium to colonize these surfaces under relevant physiological conditions. Here, we present a method in which epithelia/endothelia are simulated by flow chamber-grown human cell layers, and infection is induced by seeding of pathogenic bacteria on these surfaces under conditions that simulate the physiological microenvironment. Quantification of bacterial adhesion and colonization of the cell layers is then performed by *in situ* time-lapse fluorescence microscopy and automatic detection of bacterial surface coverage. The method is demonstrated in three different infection models, simulating *Staphylococcus aureus* endothelial infection and *Escherichia coli* intestinal- and uroepithelial infection. The approach yields valuable information on the fitness of the bacterium to successfully adhere to and colonize epithelial surfaces and can be used to evaluate the influence of specific virulence genes, growth conditions, and antimicrobial treatment on this process.

## Introduction

Historically, studies of microorganisms have been conducted on cultures that are grown in liquid broth or on agar plates. However, the traditional laboratory cultures rarely reflect the actual growth conditions in the microorganism's natural habitats. When growing in their natural habitats, both gene expression and phenotypical characteristics often differ considerably (Brock, 1971; Branda et al., 2001; Kuthan et al., 2003; Palková, 2004). This is also the situation for pathogenic bacteria that colonize and invade the human body (e.g., Smith, 1998; Krismer et al., 2014; Zapotoczna et al., 2016). Here, immune effectors, scarce nutrient availability, and hydrodynamic conditions drastically affect bacterial growth and force them to utilize alternative growth strategies and activate stress responses.

A typical response to such stressful conditions is the formation of bacterial biofilm. Bacterial biofilm has drawn much attention in recent decades, which has led to the establishment of several methods for studying this specific growth response (Lebeaux et al., 2013; Azeredo et al., 2017). Such models typically involve culturing of biofilms on abiotic surfaces such as glass or plastic, in some cases coated with specific proteins to mimic a biological surface (Djordjevic et al., 2002; Lembke et al., 2006).

The first host barrier encountered by invading bacteria is often the epithelial mucous membranes lining the body's internal tubular structures. To study the ability of an invading pathogen to adhere to this inner surface, a so-called static microtiter plate adhesion assay is typically applied. In this easy and high-throughput method, bacteria are added to adherent epithelial cell cultures followed by centrifugation to facilitate bacteria-cell contact. Lastly, adherent/invasive bacteria are quantified by colony-forming unit (CFU)-enumeration on agar plates (e.g., Berry et al., 2009; Letourneau et al., 2011). This enables assessments of functional bacteria-epithelium cell adhesion strengths. However, only the initial contact between the cells can be investigated by this method, since co-culturing in the static system leads to fast nutrient depletion, bacterial overgrowth, and degradation of the cell culture.

Recent studies have demonstrated that prolonged bacteria-epithelium infection experiments can be simulated using flow chamber based infection models. Such models facilitate investigation of bacteria-epithelium interactions under the exposure of physiological liquid shear (Andersen et al., 2012; Alsharif et al., 2015; Khandige et al., 2016; Stærk et al., 2016). They furthermore induce *in vivo*-like adhesion stimuli in bacteria as well as the possibility to study the influence of the physiological microenvironment on the mechanisms of pathogenesis (Andersen et al., 2012; Khandige et al., 2016). Although specific bacterial gene expression and phenotypical changes can be investigated in such flow models, they have not been optimized for accurate quantification of the progression of bacterial establishment and biofilm formation on epithelial cell layers. Bacteria can be extracted from flow chambers after specific periods of time for CFU determination. However, this requires termination of a rather laborious experiment, leaving only a single data point measured per chamber. Furthermore, CFU enumeration of bacteria exposed to *in vivo*-like stress conditions can be tricky, since bacteria may change morphology, i.e., become filamentous (Klein et al., 2015; Khandige et al., 2016), or aggregate (Loof et al., 2015; Grønnemose et al., 2017; Sønderholm et al., 2017), or slow down growth speed (Proctor et al., 2006), which may affect the accuracy of bacterial quantification by traditional plating techniques.

Here we present a method that circumvents these problems, allowing accurate and reproducible quantification of bacterial colonization on epithelial cell layers under physiological hydrodynamic conditions. The method is demonstrated in three infection models, simulating urinary tract infection, intestinal infection, and bloodstream infection by the important human pathogens; uropathogenic- and Shiga toxin-producing *Escherichia coli* (UPEC and STEC, respectively), and *Staphylococcus aureus*.

Bacterial colonization of flow chamber-cultured uroepithelial, intestinal epithelial, and endothelial cell layers is quantified based on fluorescence signals from adherent bacteria that constitutively express green fluorescent protein (GFP). Time-lapse microscopy and quantification of the bacterial coverage is performed at several predetermined sites on the cell layers to obtain accurate mean overall values of the growth progression as well as information about growth pattern. The method extends the standard microtiter plate-based bacteria-epithelium adhesion assay by including hydrodynamic stress conditions and continuous monitoring of bacterial surface colonization for prolonged periods of time. It furthermore opens up for detailed studies on the influence of known or potential virulence factors, immune effectors or antimicrobial treatment on the progression of infection. Here, we demonstrate this applicability in a study of the influence of the *E. coli* type 1 fimbriae (T1F), on UPEC adhesion/colonization capacity on uroepithelium cell layers in a flow of artificial urine. Although the T1F tip adhesin FimH is well known to interact with uroplakin on the uroepithelial cell surface and promote adhesion/invasion in the urinary tract (Zhou et al., 2001; Bouckaert et al., 2006), its influence on bacterial colonization of the uroepithelium has to our knowledge not been quantified directly in a continuous monitoring *in vitro* setup. The current method uniquely allows testing of this under relevant physiological conditions.

## Materials and methods

### Bacteria, cells, and growth conditions

#### Intestinal infection: shiga toxin-producing *Escherichia coli* colonization of intestinal cell layers (T84)

Flow chamber-cultured layers of T84 cells (ATCC CCL-248) were used to model the human intestinal epithelium. The T84 cell line is an immortal intestinal epithelial cell line derived from a lung metastasis of a patient with colon carcinoma. T84 cells were subcultured in T25 flasks (Nunc, Easy Flask, Delta Surface) at 37°C in a humidified atmosphere with 5% CO_2_ using Dulbecco's Modified Eagle Medium (DMEM)/F-12 with GlutaMAX^TM^ (Gibco) supplemented with 5% fetal bovine serum (FBS) (Sigma) and 1% Penicillin-Streptomycin (PS) (Stock: 10.000 Units/ml Penicillin, 10.000 μg/ml Streptomycin, Gibco) as growth medium. Experiments were conducted using T84 in passage 57-77. Cells were liberated from culture flasks using Trypsin-EDTA (Sigma), resuspended in 5 ml cell media, of which 150 μl was added to the flow chambers (1 μ-Slide I^0.6^Luer Collagen IV, Ibidi, Germany). Seeded cells were allowed to settle for 12 h before adding new growth media. Growth medium was changed every 24 h until cells reached <95% confluence, typically within 6–7 days.

STEC strain EDL933 was used as model intestinal pathogen. EDL933 is an isolate of the serotype O157:H7 originally cultured from Michigan ground beef and associated with a multistate outbreak of hemorrhagic colitis in the US (Riley et al., 1983; kindly provided by Dr. T. Shimizu). All experiments with this strain were conducted in facilities licensed by the Centre for Biosecurity and Biopreparedness according to the Danish biosecurity law (Act no. 474, 2008).

Green fluorescent EDL933 was produced by transformation with the pMAN01 plasmid, containing a chloramphenicol resistance gene. The pMAN01 plasmid was constructed by ligating an EcoRV-SapI restriction fragment containing a transcriptional fusion of the *gfp*+ gene to the hsp60 promoter, derived from pMN402 (Scholz et al., 2000), into pBAD33. The resulting plasmid encodes for strong constitutive expression of the GFP+ reporter protein. Bacteria were grown in Luria broth (Invitrogen, Miller's LB Broth Base) supplemented with 30 mg/l chloramphenicol (Sigma-Aldrich) at 37°C overnight (ON) in a 2-day regimen without shaking. In brief, 10 μl of stationary phase culture were added to a new vial after one night of incubation, and subsequently the second ON culture was used as seeding suspension in the flow chambers. For STEC growth in the flow chambers, a modified M9 minimal medium was formulated according to Elbing and Brent (2002) with the following components added to each liter of distilled water: 6.0 g disodium phosphate, 3.0 g monopotassium phosphate, 1.0 g ammonium chloride, 0.5 g sodium chloride, 2.0 g D-glucose, 1.0 g casein hydrolysate, 0.5 mg thiamine hydrochloride (all from Sigma-Aldrich), 4.0 mg calcium chloride·2H_2_O, 0.228 g magnesium sulfate·6H_2_O (both from Riedel-de Häen AG). The mixture was dissolved, and pH adjusted with 5 M hydrochloric acid to 6.6 (similar to feces; Rose et al., 2015) and sterile filtered at 0.2 μm before use.

#### Disseminated bloodstream infection: *Staphylococcus aureus* colonization of endothelial cell layers (EA.hy926)

Flow chamber-cultured layers of the human endothelial cell line EA.hy926 was used to model the endothelial surface, and *Staphylococcus aureus* ATCC 29213 was used as a model bloodstream pathogen. The EA.hy926 (ATCC CRL-2922) endothelial cell line is an immortalized fusion of a human umbilical vein endothelial cell (HUVEC) and a human pulmonary adenocarcinoma A549 cell. EA.hy926 cells were cultured in DMEM containing 4.5 g/l D-Glucose, 584 mg/ml L-glutamine, 110 mg/l Sodium pyruvate (Gibco) and supplemented with 10% FBS, and 1% PS in T25 cell culture flasks at 37°C with 5% CO_2_. EA.hy926 cells were liberated from the bottom by trypsination and sub-cultured when reaching 80% confluence. 150 μl of a 5 ml cell split were reseeded in the flow chamber slides (μ-Slide I^0.6^Luer Collagen IV, Ibidi, Germany). For the EA.hy926 cells to reach 100% confluence, the chamber cultures was incubated for 2 days at 37°C with 5% CO_2_. On the second day, the chambers were connected to a peristaltic pump and exposed to a continuous flow of cell medium (DMEM/10% FBS) at 92 μl/min until the next day to allow the cell layer to adapt to flow conditions and wash out antibiotics. For all experiments, a variant of the *S. aureus* strain ATCC 29213 was used which constitutively expresses GFP under the control of the staphylococcal blaZ-promotor (generously provided by Dr. Oleg Krut Schnaith et al., 2007). Prior to infection, *gfp*-transformed *S. aureus* ATCC 29213 were grown in sterile filtered tryptic-soy broth (TSB, Sigma) containing 30 mg/l chloramphenicol for 2½ h at 37°C with shaking to reach exponential phase, at which the expression of adhesins are elevated (Novick, 2003). Bacteria were pelleted by centrifugation at 2000 *g* for 2 min and resuspended in 10% heparinized human plasma for 10 min. This step was performed to coat the bacteria with human clotting factors and other plasma proteins thus simulating *in vivo* opsonization/protein adsorption when *S. aureus* enters the bloodstream. The suspension was then once more centrifuged at 2000 *g* for 2 min and re-suspended in 0.9% NaCl buffer to an OD_600 nm_ of 0.02. A 700 μl aliquot was transferred to 69.3 ml DMEM/10% FBS and used directly as seeding suspension corresponding to a total inoculum of approximately 1.4 × 10^7^ CFU.

#### Urinary tract infection: uropathogenic *Escherichia coli* colonization of uroepithelial cell layers (HTB9)

As uroepithelial cells, the ATCC HTB9 cell line was used, subcultured in T25 flasks at 37°C in a humidified atmosphere with 5% CO_2_ using Roswell Park Memorial Institute (RPMI) 1640 medium (Gibco) supplemented with 10% FBS and 1% PS as growth medium. The ATCC HTB9 uroepithelial cell line is an immortal cell line derived from a human bladder carcinoma. As the model UPEC, the *Escherichia coli* strain UTI89 was used, a cystitis-derived isolate of serotype O18:K1:H7 previously used in several *in vitro* and *in vivo* urinary tract infection (UTI) model studies (Mulvey et al., 2001; Justice et al., 2004, 2006). The UTI89Δ*fimH* deletion mutant, was constructed as reported previously (Andersen et al., 2012). Green fluorescent variants of UTI89wt and UTI89Δ*fimH* were constructed by transforming with the pMAN01 plasmid as described above for STEC EDL933. For UTI89 pre-culturing and growth in the flow chambers, artificial urine (AU) was used in a formulation according to Brooks and Keevil (1997). In short, the following components were added to each liter of distilled water: 1.3 g ammonium chloride, 0.4 g citric acid, 2.1 g sodium bicarbonate, 5.2 g sodium chloride, 10 g urea, 5 mg select yeast extract (all from Sigma-Aldrich), 0.37 g calcium chloride·2H_2_O, 0.454 g magnesium sulfate·6H_2_O (both from Riedel-de Häen AG), 3.2 g sodium sulfate·10H_2_O, 1.2 g dipotassium hydrogen phosphate (both from Merck), 0.454 g iron II sulfate·6H_2_O, 92 μl lactic acid (both from VWR), 0.8 g creatinine (Alfa Aesar), 1 g peptone L37 (Oxoid), 0.95 g potassium dihydrogen phosphate (Fluka Chemika), and 0.07 g uric acid (AppliChem). The mixture was adjusted to pH = 6.4 with 5 M hydrochloric acid and sterile filtered before use. We recommend storing the solution in plastic containers since storage in glass containers tends to trigger precipitation of the solution, for unknown reasons. Before seeding in flow chambers, UTI89 was pre-cultured in a 2-day regimen in either Luria Broth (to stimulate T1F expression) or AU (to simulate physiological conditions). An initial ON culture were prepared by inoculating an agar plate colony in 35 ml of the specific medium. The subsequent day, 10 μl were transferred to a new 35 ml medium vial and incubated ON.

### Flow chamber infection of cell layers

Flow chamber infections were performed in commercial flow chambers of the type Ibidi μ-slide^0.6^ (Ibidi, Germany). Bacterial strains used for the experiments are shown in Table 1.

**Table 1:** Strains used in the study.

**Strain**	**Type**	**Description**	**GFP plasmid**	**Source**	**Reference**
EDL933	Shiga toxin-producing *E. coli* O157:H7	Meat isolate implicated in an outbreak of hemorrhagic colitis	pMAN01	Dr. T Shimizu	Riley et al., 1983
ATCC29213	*S. aureus* subsp. aureus Rosenbach	Wound isolate	pS1-GFP	Dr. O Krut	Schnaith et al., 2007
UTI89	Uropathogenic *E. coli* O18:K1:H7	Cystitis isolate	pMAN01	Prof. DJ Klumpp	Mulvey et al., 2001
UTI89Δ*fimH*	UTI89 deleted in the *fimH* gene	–	pMAN01	Own construct	Andersen et al., 2012

In experiments with T84 and HTB9 cells, cells were grown to confluence in flow chambers under static conditions as described above. Chambers were then connected to a peristaltic pump (Ismatec IPC-N8, Glattbrugg, Switzerland) through sterile silicone tubes, and a constant flow of 200 μl/min of complete growth medium was applied for 2 days at 37°C and 5% CO_2_. Upon application of flow, approximately 50% of the cells are washed off and slowly replaced by a more flow-resistant cell layer that ultimately reaches >95% confluence within 2 days. Prior to infection, the flow chambers were disconnected from the pump, and cells were washed 10 times with Hank's balanced salt solution supplemented with calcium chloride and magnesium chloride (HBSS, Gibco) to remove any proteins derived from the medium. Cells were then fixed by incubating without flow for 1 h in 10% neutral buffered formalin solution (Sigma, product number HT-5011), at 37°C and 5% CO_2_ and subsequently washed for another 10 times in HBSS to remove formalin from the flow chambers. Flow chambers were then reconnected to the pump, and any remaining formalin was washed out at 200 μl/min for 30 min in the flow-media used for the subsequent infection experiments. Cell layers were then infected by perfusion of the flow chambers with bacteria suspended in phosphate-buffered saline (PBS) (SSI diagnostica) to an optical density (OD_600_) of 0.2 corresponding to approximately 1.6 × 10^8^ CFU/ml. The seeding suspension was passed through the chambers at 100 μl/min for 20 min as previously described (Stærk et al., 2016). Then, selective media was connected to the chamber (modified M9 and AU, respectively with 30 mg/l chloramphenicol for pMAN01 plasmid stability) and adherent bacteria allowed to colonize the cell layer for 24 h at flow rates of 50 μl/min (modified M9) and 200 μl/min (AU) for STEC and UPEC isolates, respectively.

In *S. aureus* infection of EA.hy926 cell layers, bacterial seeding suspension (see above) was passed through the chamber for 10 min at a flow rate of 6.4 ml/min. Following, new sterile tubes were connected to the chamber, and for the remaining time of the experiment, sterile DMEM with 10% FBS was pumped through the chambers at a sequential flow regime consisting of 1 min at 6.4 ml/min flow interrupted by 9 min pause, restarting every 10 min.

### Quantification of surface colonization by image analysis

Colonization of the flow chambers was monitored by automated time-lapse fluorescence and phase-contrast microscopy. Using a BX51 microscope and Olympus cellSens software (version 1.7), 11 separate positions in each flow chamber were randomly selected, comprising a total surface area of 10.23 mm^2^. These positions were then assigned to the cellSens Experiment Manager, and the software was programmed to sequentially capture fluorescent images (GFP) and phase-contrast images of all 11 positions every 20 min for a predetermined number of cycles. After completion of time-lapse recording, the fluorescent images were analyzed by the cellSens Count and Measure tool using interval gates to both capture the fluorescent bacteria and exclude background signal. Coverage in percentage was defined as Region of Interest (ROI) values. Graphs over the progression of bacterial colonization represent mean values from 3 to 4 separate flow chamber experiments ± one standard deviation (SD).

### Statistical analysis

The Wilcoxon rank-sum test was used to compare ROI values of UTI89wt pre-cultured in LB with both UTI89wt pre-cultured in AU and UTI89Δ*fimH* pre-cultured in LB. Using the Bonferroni correction of 8 comparisons, *p* < 0.006 (0.05/8) were considered statistical significant. The 95% confidence intervals (CI) were obtained using bootstrap analyses of 10^5^ replicates. All analyses were performed in STATA version 15.0 (StataCorp LLC, College Station, TX, USA).

## Results

### Experimental setup

**Figure 1:**
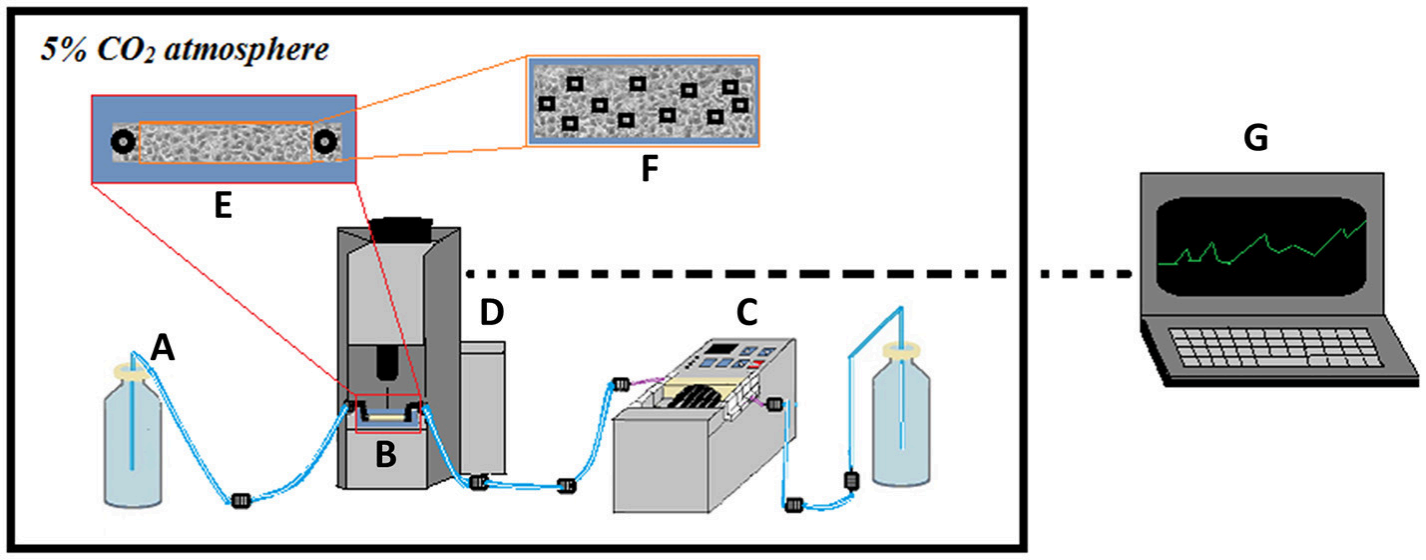
Scheme showing the experimental setup used for studying bacterial colonization of cell layers. A once-through flow-setup is applied with sterile media **(A)** pumped through the flow chamber **(B)** using a high-precision peristaltic pump located downstream of the flow chamber **(C)**. The flow chamber is placed on a computer-controlled, motorized stage under the microscope **(D)**, allowing image recording of the infected cell layer **(E)**. Predetermined locations on the cell layer **(F)** are monitored by time-lapse phase-contrast and fluorescence microscopy, with data extracted subsequently and computer-processed into growth curves **(G)**. The entire setup is kept in an incubator to control temperature and CO_2_ levels.

Figure 1 shows a sketch of the experimental setup. For optimal cell culturing conditions, the entire setup is placed in a CO_2_ incubator. Flow chambers are placed on a high-precision, motorized, computer-controlled stage under the microscope. Image acquisition is configured to capture images every 20 min at 11 preset locations in the flow channel to obtain representative mean values of the progression of colonization. After seeding with bacteria, sterile, once-through flow is applied in order to specifically monitor the ability of the initially seeded bacteria to increase in numbers on the surface under fluid flow conditions.

In the current experiments, data were recorded over time periods of 24–26 h and include measurements of number and area of individual microcolonies as well as total coverage in percent (ROI-%). Based on parallel CFU-quantifications during the initial phases of infection, the detection limit was estimated to approximately 10^6^ bacteria per chamber, and only clustered bacteria at the microcolony-stage is detected.

### STEC colonization of intestinal epithelial cell layers

**Figure 2:**
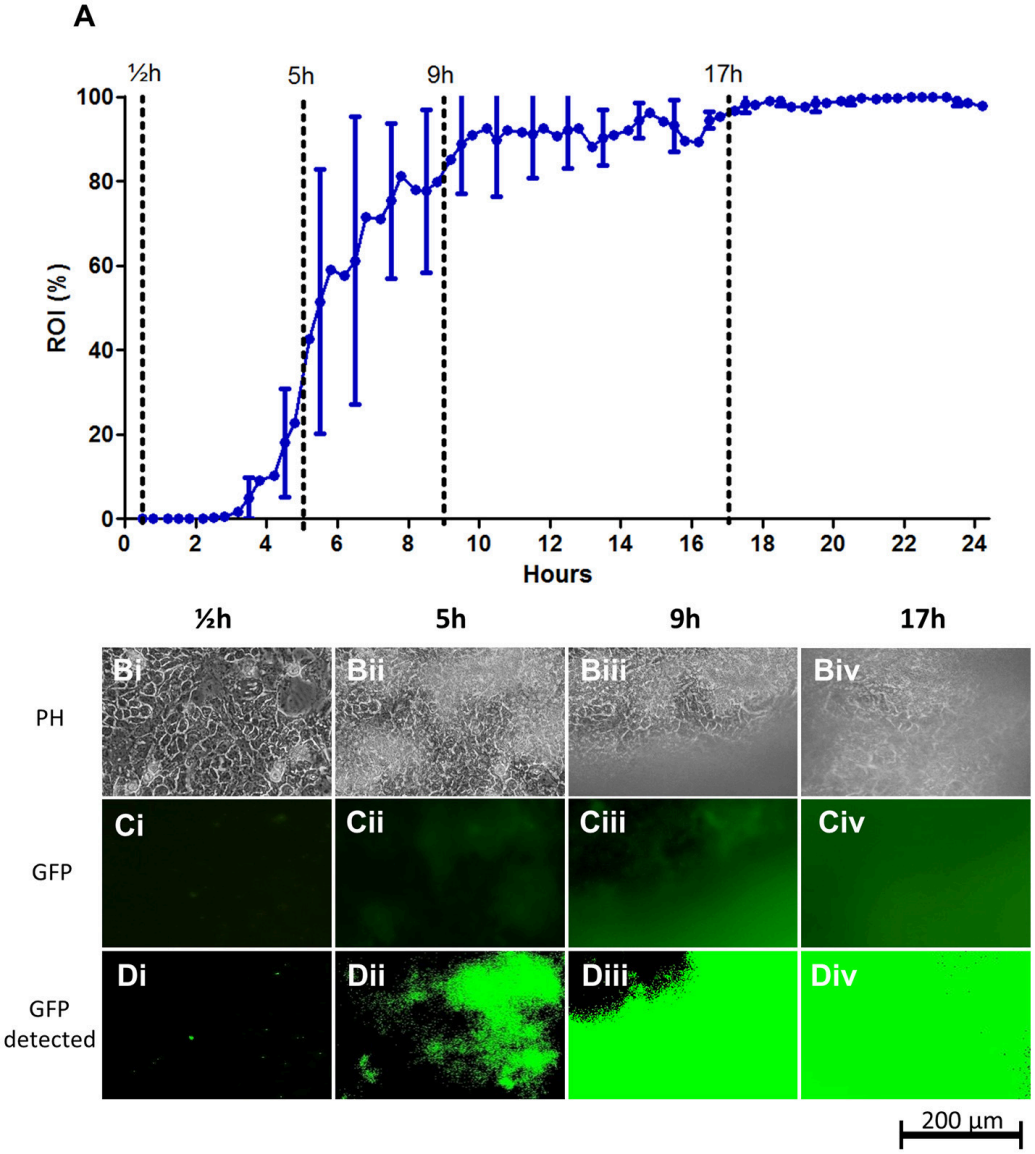
STEC strain EDL933 colonization on fixed T84 intestinal epithelial cell layers under flow. **(A)** Graph showing the progression of bacterial growth on the surface. The data points represent mean values of the bacterial coverage in percent at 20 min intervals from four separate flow chamber runs. Error bars represent ±1 SD between individual flow chamber runs (shown, for clarity, only at 1-h intervals). Values at each 20-min time point obtained in each flow chamber run are mean values from recordings at 11 positions each representing 1,100 × 850 μm on the cell layer surface. The graph is based on a total of 2,684 single scans. **(B–D)** Examples of image data recorded at one representative 380 × 250 μm cropped position. Phase contrast microscopy **(Bi–Biv)**, fluorescence microscopy **(Ci–Civ)**, and software-detected coverage **(Di–Div)** are shown at four specific time points. These time points are marked with broken lines in **(A)** representing four phases of surface colonization; seeding (½h; **Bi**,**Ci**,**Di**), initial establishment on the surface (5 h; **Bii**,**Cii**,**Dii**), stabilization on the surface (9 h; **Biii**,**Ciii**,**Diii**), and steady state (17 h; **Biv**,**Civ**,**Div**). GFP, green fluorescent protein; h, hours; PH, phase contrast microscopy; ROI, region of interest.

Intestinal colonization by the prototypical STEC serotype O157:H7 strain EDL933 was modeled using cell layers of the T84 cell line (Figure 2). Live T84 cells were found to destabilize quickly when infected with EDL933, probably resulting from Shiga toxin secretion by EDL933. Hence in this infection model, formaldehyde-fixed T84 cell layers were used as substratum. As growth medium, a simple M9-based medium supplemented with glucose and casein was used. No growth medium ideally reflects feces due to its highly heterologous composition. Instead we chose this relatively neutral medium which does not contain any irrelevant animal- or yeast-derived components. At the same time, this medium is highly transparent, ensuring a high signal-to-noise ratio when recording fluorescence signals from the chamber.

After a lag time of approximately 3 h post infection (hpi), growth of EDL933 on the T84 cell layers increased rapidly up to an ROI-value of approximately 50% at 6 hpi. As shown in Figures 2B–D, growth initiates as sporadic microcolonies dispersed on the T84 cell layer that slowly increase in size, finally acquiring a biofilm-like appearance. From 6 hpi onwards, larger clumps of biofilm material occasionally wash off from the cell layer, leading to brief drops in ROI percentage during the experiment and larger SD-values (Figure 2A, Figure S1). This shedding of biofilm material temporarily slows down growth until 17hpi from where growth stabilizes near 100% coverage.

The sporadic growth on the surface gave rise to considerable deviation in biofilm coverage between microscopy images acquired during the growth phase of the biofilm (individual flow chamber runs with SD error bars indicating the deviation between individual captured images are shown in Figure S1). This emphasizes the need for the retrieval of mean values from several images in each flow chamber for optimal accuracy. Curve paths of mean values from individual runs were relatively similar between experiments, demonstrating the reproducibility of the method (Figure S1 and indicated by the SD-error bars in Figure 2A).

### *Staphylococcus aureus* colonization of endothelial cell layers

Metastatic bloodstream infection results when invading bloodstream pathogens spread through the vascular system and reach distant endothelial or endocardial sites. Here, the pathogen manages to adhere firmly to the endothelium/endocardium despite considerable liquid shear, followed by proliferation and invasion of underlying tissue (Edwards et al., 2010). Here, we established an experimental protocol, based on the fluorescence microscopy-quantification setup, to monitor and quantify adhesion to, and initial colonization of endothelial cell layers by *S. aureus* under fluid flow. As a model bloodstream pathogen, the *S. aureus* ATCC strain 29213 was used and the vessel wall/heart valve surface was simulated using flow chamber cultured EA.hy926 endothelial cell layers. Prior to infection, *S. aureus* ATCC 29213 was treated briefly with human blood plasma to stimulate the opsonization and plasma protein coat that forms *in vivo* (Ko et al., 2013; Claes et al., 2014). As a flow medium, DMEM in 10% FBS was used to ensure a source of blood proteins including fibronectin, which is important for *S. aureus*-endothelium interaction (Edwards et al., 2010). Using this medium furthermore allowed the experimental infection to be performed as a co-culture with live endothelial cell layers throughout the experiment.

Simulation of vascular wall shear stress requires high flow rates. In the current type of infection experiment, such a shear stress cannot be generated by recirculation of the media, since it would quickly lead to uncontrolled and continuous seeding of bacteria in the chamber as well as rapid depletion of nutrients and accumulation of waste products in the circulating growth media. On the other hand, using continuous once-through flow at high flow rates inevitably leads to an enormous consumption of expensive media in prolonged infection experiments. As a compromise, we chose to use a sequential, once-through flow consisting of 1 min of high flow (6.4 ml/min) yielding a wall shear stress of 2.77 dynes/cm^2^, interrupted by a pause of 9 min, followed by cycle restart. The 2.77 dynes/cm^2^ shear stress is within the rates found in the large venous and arterial circulation (approximately 1 to 12 dynes/cm^2^; Papaioannou and Stefanadis, 2005). Despite periods of time without flow, the occasional high-flow sequence ensures that the bacteria are still forced to establish contact to the surface with the same force of adhesion, as if continuous flow is used.

**Figure 3:**
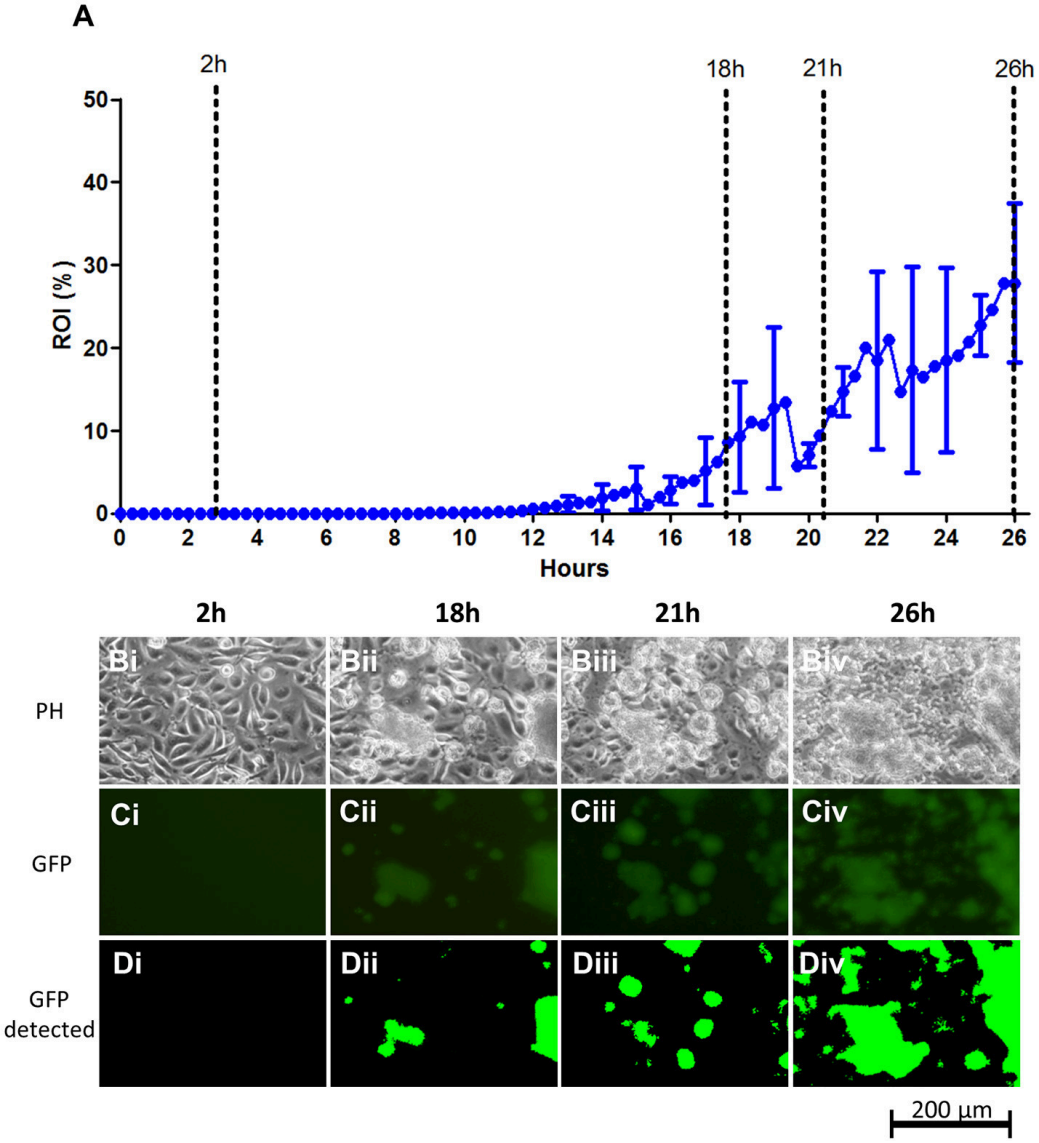
*S. aureus* ATCC 29213 colonization on live EA.hy926 endothelial cell layers under flow. **(A)** Graph showing the progression of bacterial growth on the surface with data points representing the mean values of the bacterial coverage in percentage at 20 min intervals from three separate flow chamber runs. Error bars represent ±1 SD between individual flow chamber runs (shown, for clarity, at 1 h intervals). Values at each 20 min time point obtained in each flow chamber run are mean values from recordings at 11 positions each representing 1,100 × 850 μm on the cell layer surface (in this graph, data are based on a total of 2,607 single scans). **(B–D)**. Example of image data recorded at one position, representing a cropped 380 × 250 μm area with above average growth. Phase contrast- **(Bi–Biv)**, fluorescence microscopy **(Ci–Civ)**, and software-detected coverage **(Di–Div)** are shown at four specific time points (marked with broken lines in **A**). GFP, green fluorescent protein; h, hours; PH, phase contrast microscopy; ROI, region of interest.

When applying this experimental protocol to study and quantify the adhesion and colonization of *S. aureus* on the endothelium, we found that the bacterium exhibited a considerable delay in initiation of surface colonization. As shown in Figure 3, the first bacterial colonies are detected 12–15 hpi. The delay in surface colonization is similar between runs; however, growth curves from individual flow chamber runs show occasional large drops in overall coverage in each chamber, resulting from release of large biofilm clumps after they build up on the surface (for individual runs, see the graphs in Figure S2). Even more pronounced for *S. aureus* compared to STEC, is the sporadic growth, which occurred only from a relatively few sites on the endothelial surface (data not shown).

*S. aureus* is known to be highly invasive and it is likely that some extent of intracellular colonization is present in the live EA.hy926 cell layer, also during the initial phase. Such signals are, however, not picked up by the microscope camera setup, possibly due to the detection limit of approximately 10^6^ CFU/chamber.

### UPEC colonization of uroepithelial cell layers

In previous studies we investigated the interaction of UPEC with uroepithelial cell layers in flow systems (Khandige et al., 2016; Stærk et al., 2016). This previous approach enabled qualitative *in situ* microscopy analyses of bacterial phenotypes on the cell layers. However, it lacked the opportunity to quantify the bacterial capacity to adhere to and colonize the cell layer surface when exposed to significant liquid shear. Here, we applied the fluorescence-based quantification method to obtain such measurements. Artificial urine (AU) was used as flow medium at a flow rate of 200 μl/min corresponding to a wall shear stress of 0.08 dynes/cm^2^ (shear rate = 12.02 s^−1^). This is approximately six times higher than the value used in earlier studies (2 s^−1^, Andersen et al., 2012; Khandige et al., 2016; Stærk et al., 2016), and applied in the current model to let adhesion strength be a main limiting factor for colonization, thus enabling discrimination of UPEC with adhesion/colonization deficiencies. The uroepithelial cell layers in the model demonstrated here were fixed prior to infection to retain a completely confluent cell layer during infection and to monitor adhesion and surface colonization without the influence of cellular invasive events.

**Figure 4:**
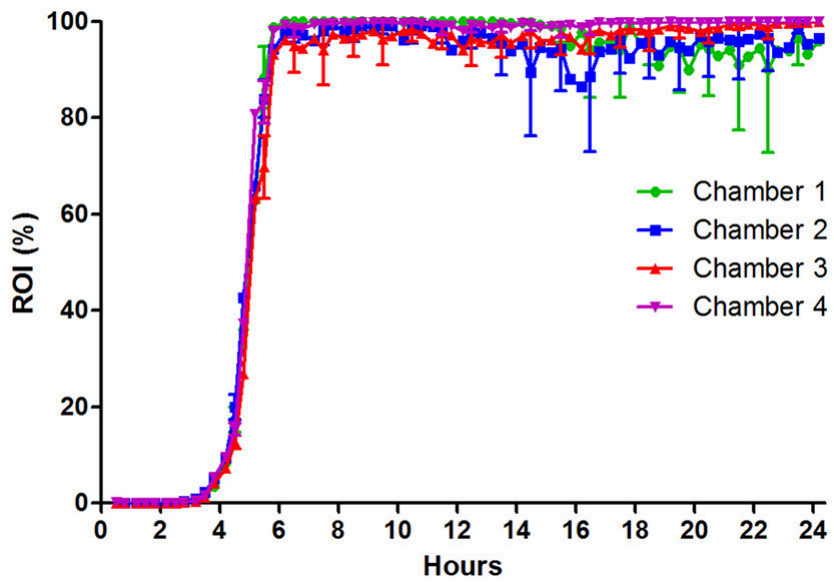
UPEC strain UTI89 colonization on fixed HTB9 uroepithelial cell layers under flow of artificial urine medium. Four individual flow chamber runs are shown together to visualize the high degree of reproducibility from experiment to experiment. Eleven 1,100 × 850 μm sites were monitored in each flow chamber over 24 h. Error bars indicate ±1 standard deviation. ROI, region of interest.

Similar as for EDL933, UTI89 exhibits an initial lag-phase (Figure 4). However, as soon as UTI89 initiates growth, coverage increases at a high exponential rate until approaching 100%. Contrary to both *S. aureus* ATCC 29213 and STEC EDL933 biofilms, the UPEC UTI89 biofilm was observed to be highly stable with loss of biomass mainly as the planktonic form of single bacteria rather than biofilm clumps (based on inspection of time lapse microscopy of the biofilm development). Due to effective seeding a relatively evenly distributed growth of UPEC was observed on the entire surface, giving rise to little variation between images captured in individual flow chamber runs (indicated by SD error bars in Figure 4). Variation between individual flow chamber runs was very low, showing good reproducibility.

### Assessment of the role of type 1 fimbriae-mediated adhesion in UPEC colonization of uroepithelial cell layers

T1F and its tip adhesin FimH are among the most comprehensively studied virulence factors in UPEC and believed to contribute to uroepithelial adhesion and colonization of the human urinary tract. Studies have demonstrated that T1F facilitates adhesion to human uroepithelial cells and increases virulence *in vivo*, based on static cell adhesion assays and mouse infection models (Connell et al., 1996; Wright et al., 2007). However, a reported low expression of the *fim* genes *in vivo* has raised questions about the regulation and importance of T1F during UTI (Lim et al., 1998; Hagan et al., 2010).

**Figure 5:**
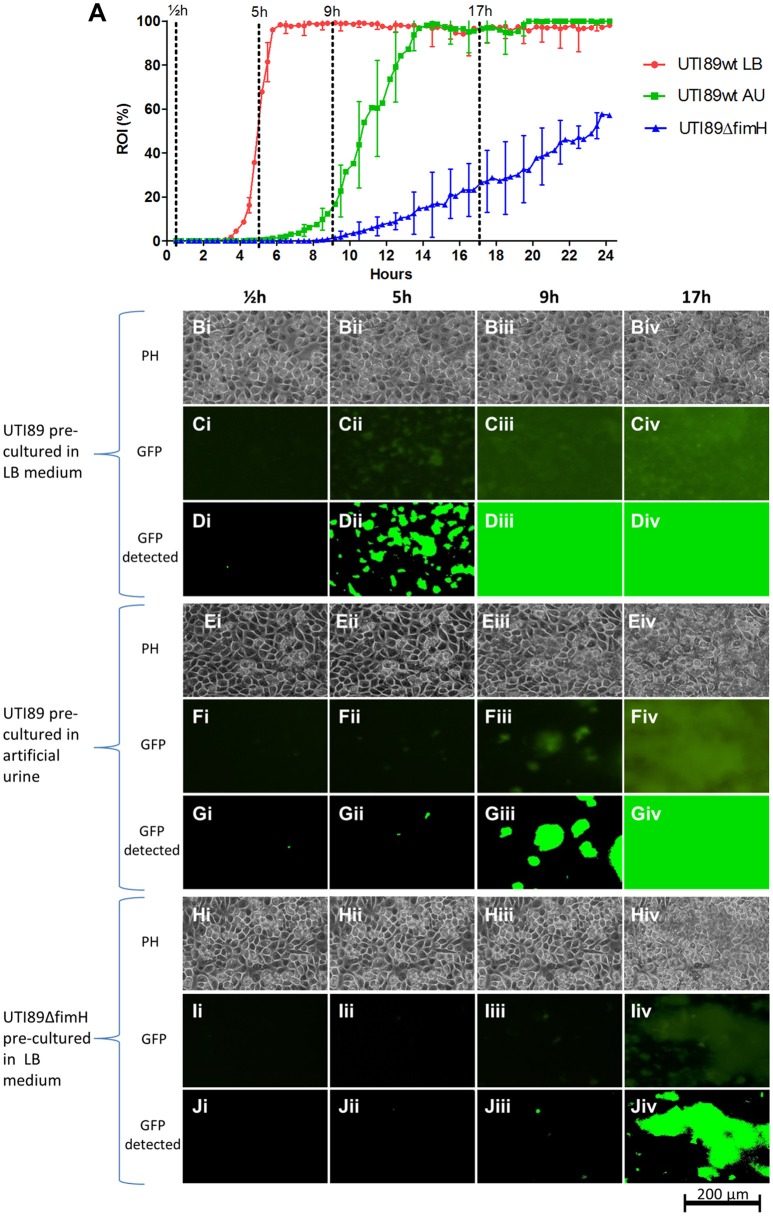
UPEC UTI89 colonization on fixed HTB9 uroepthelial cell layers under flow. **(A)** Graph showing the progression of bacterial growth on the surface by UTI89wt pre-cultured in LB (red line), UTI89wt pre-cultured in AU (green line) and UTI89Δ*fimH* pre-cultured in LB (blue line). The data points represent mean values of the bacterial coverage in percent at 20 min intervals from three separate flow chamber runs. Error bars represent ±1 SD between individual flow chamber runs (shown, for clarity, at 1-h intervals). Values at each 20 min time point obtained in each flow chamber run are mean values from recordings at 11 positions each representing 1,100 × 850 μm on the cell layer surface (in the graph, data is compiled from a total of 8,640 single scans). **(B–D)** Example of image data recorded at one representative 380 × 250 μm cropped position. Phase contrast (**Bi–Biv,Ei–Eiv,Hi–Hiv**), fluorescence microscopy (**Ci–Civ,Fi–Fiv,Ii–Iiv**), and software-detected coverage (**Di–Div,Gi–Giv,Ji–Jiv**) are shown at four specific time points. These are marked with broken lines in **(A)** representing different phases of surface colonization. Coverage by UTI89wt precultured in LB and AU differ significantly, at 5 and 9 h post seeding (*p* < 0.001, no overlap of the 95% CI). Coverage by UTI89wt and UTI89Δ*fimH* differ significantly at 5, 9, and 17 h post seeding (*p* < 0.001, no overlap of the 95% CI). GFP, green fluorescent protein; h, hours; PH, phase contrast microscopy; ROI, region of interest.

To assess the effect of T1F-mediated adhesion on the colonization capacity of UTI89 on uroepithelium under conditions that reflect the urinary tract milieu, a UTI89 strain deleted in the *fimH* gene (lacking functional T1F), was analyzed using the current method and compared to UTI89wt/pMAN01. This result showed a drastic influence of T1F on the ability of UPEC to establish contact to and colonize the uroepithelium (Figure 5). The onset of colonization by UTI89Δ*fimH* is delayed approximately 6 h compared to UTI89wt (LB pre-cultured) and once growth is initiated, UTI89Δ*fimH* exhibits an approximately 18 times longer surface area doubling time with final coverage reaching only approximately 57% after 24 h (Figure 5 and Table 2).

**Table 2:** Mathematical models of the biofilm build-up during exponential phase.

**Strain and preculture**	**Exponential phase slope**	**ROI doubling time**	**R^2^**
UTI89wt in LB	e^2.339t^	18 min	0.991
UTI89wt in AU	e^0.768t^	54 min	0.997
UTI89Δ*fimH* in LB	e^0.128t^	325 min	0.980

*Slopes and the coefficient of determination (R^2^) of best fit lines are shown. Time (t) is measured in hours. LB, Luria Broth; AU, artificial urine; ROI, region of interest*.

In previous studies, we and others have demonstrated that UTI89 and other UPEC strains grown planktonically in urine exhibit very low T1F expression (Lim et al., 1998; Roos et al., 2006; Reisner et al., 2014; Greene et al., 2015; Stærk et al., 2016). We found in this earlier study that the low level of T1F-positive bacteria in urine did not significantly affect the ability of UPEC to establish a biofilm on catheter material (Stærk et al., 2016). Here, we wished to assess whether the low level of T1F-positive UPEC in urine affected the ability of UPEC to initiate surface colonization on uroepithelium.

UTI89wt was pre-cultured in artificial urine (AU) leading to mainly T1F-negative bacteria similar to culturing in real urine (data not shown). These bacteria were then seeded on HTB9 cell layers in the flow setup and allowed to colonize the surface in a flow of AU. Figure 5A shows that the AU-precultured UTI89 is inhibited considerably in its ability to colonize the uroepithelium surface, with delayed initiation of colonization and 3 times longer area doubling time once growth has entered exponential phase (Table 2). Eventually, the AU pre-cultured population gains a foothold and finally reaches approximately 100% surface coverage after approximately 14 h.

Table 2 displays the slopes of best-fit functions describing the growth curves for the three UPEC preparations analyzed in Figure 5A (see Figure S3 for lines fitted to ln-transformed data). The difference in colonization kinetics reflects different limitations in the ability of the bacterium to increase its surface-associated biomass under the applied conditions. The fast area doubling times for UTI89wt pre-cultured in LB is probably mainly limited by its speed of reproduction, since daughter cells are to a large extend retained on the surface through tight T1F-mediated surface-anchoring. The result is a growth curve reminiscent of ordinary broth-culturing. The colonization and biofilm build-up displayed by the UTI89Δ*fimH* is, on the other hand, near counterbalanced by extensive loss of daughter cells to the flow, resulting in very slowly increasing surface-associated biomass.

### Bacterial seeding efficiency

The ability of a bacterium to efficiently establish a foothold on a given surface under the exposure to liquid shear stress depends on its ability to initially adhere to the surface as well as the ability of adherent bacteria to proliferate on the surface (colonization). The latter depends on a balance between proliferating on the surface and the loss of bacterial cells to the flow.

**Figure 6:**
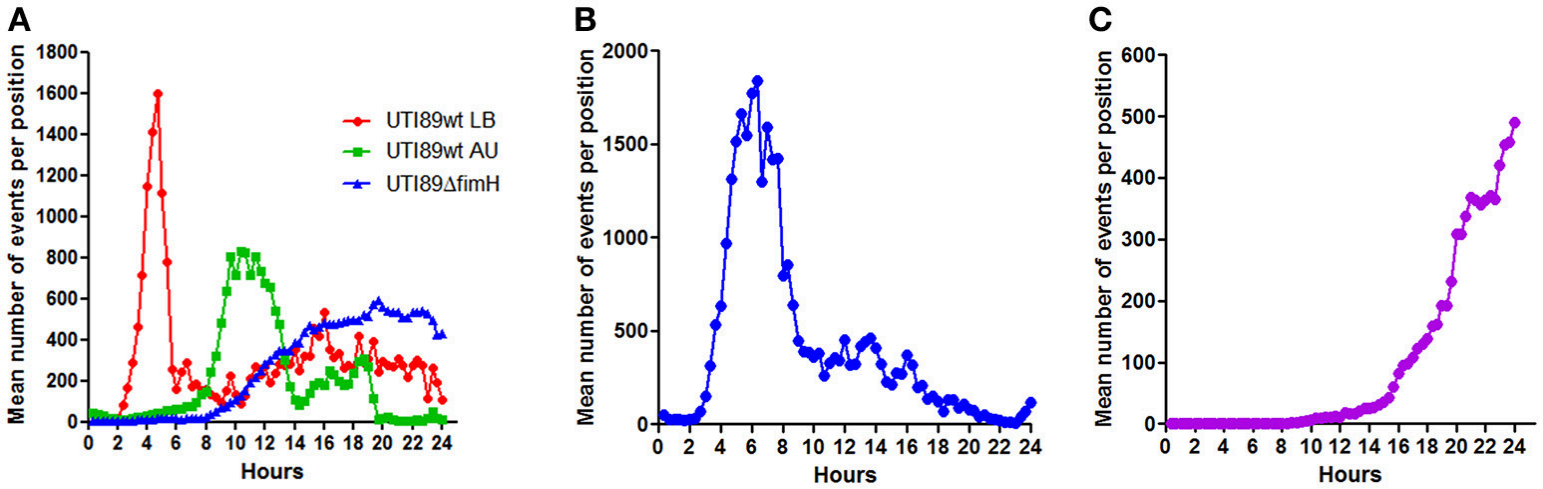
Bacterial seeding efficiency. Graphs showing the mean, total number of microcolonies (events) detected at 1,100 × 850 μm sub-locations at each 20-min time point during infection experiments with three different pathogens. Data for each type of experiment were pooled from all conducted flow chamber experiments. Peaks indicate the maximum number of bacteria which succeed in establishing a microcolony after initial seeding. **(A)** UPEC strains UTI89wt and UTI89Δ*fimH* grown on uroepithelial cell layers in a flow of artificial urine (AU). UTI89wt was precultured in either AU or LB prior to the experiment, as indicated. **(B)** STEC growth on intestinal epithelial cells (T84). **(C)** *S. aureus* colonization on endothelial cell layers (EA.hy926).

Since the microscopic monitoring of growth in the presented setup counts single events (microcolonies), the recorded data can be used to assess how efficient a given bacterium adheres to and forms microcolonies on the surface at the initial phase. Figure 6 shows the number of events counted during the growth phases of the different bacteria and bacterial preparations analyzed in the current study. UTI89wt pre-cultured in LB showed very efficient seeding, reaching mean values of more than 1600 microcolonies per monitored site shortly after seeding, a number which subsequently drops due to the merging of the colonies (Figure 6A). The T1F-negative UTI89wt pre-cultured in AU only reached approximately half the number of adherent microcolonies at its peak compared with the UTI89wt pre-cultured in LB. The lower number of microcolonies formed initially and the delay in growth initiation explains the slower colonization rate of the UTI89wt under the applied conditions. The surface-associated biomass generated by UTI89Δ*fimH* arises from an even lower number of initially adherent bacteria. Furthermore, these exhibit a slower growth increase, probably arising from extensive loss of daughter cells to the flow, resulting in a poor overall seeding efficiency during biofilm establishment.

The STEC strain EDL933 established approximately the same number of microcolonies as did the UTI89wt pre-cultured in LB, but peaked at a later time point post-infection. Furthermore, high numbers of detected microcolonies were maintained for a longer period of time compared to the UTI89wt pre-cultured in LB. This indicates the difficulties encountered by this bacterium to form stable biofilms with merging of microcolonies under the applied conditions. Overall STEC EDL933 displayed an intermediate seeding efficiency. Only few *S. aureus* ATCC29213 bacteria were able to adhere and initiate proliferation under the applied high-flow conditions (Figure 6C), with relatively few colonies located too far apart to effectively merge into a biofilm within the 24 h time period.

## Discussion

Irreversible attachment and proliferation on host barrier epithelial surfaces is a critical first step in the pathogenesis of many infectious diseases (Finlay and Falkow, 1997; Pizarro-Cerdá and Cossart, 2006; Ribet and Cossart, 2015). Invasion at mucosa sites requires that the bacterium adheres firmly to the superficial cell layers and increases in numbers while resisting the continuous movement of fluids at these sites. Accurate *in vitro* modeling of this process requires a system that allows interaction of bacteria with relevant human epithelial cell layers under controlled hydrodynamic conditions and in relevant media.

Studies of bacteria-epithelial cell adhesion are typically conducted with microtiter plate cultured cell lines (Albert et al., 2000). However, in recent years, adhesion studies conducted in flow devices have started to gain ground. Such assays allow more control over liquid shear stress as well as monitoring of the adhesion process by microscopy. An area, in which this approach has found increasing use, is in the analysis of the interaction between bloodstream pathogens and endothelial cells. Here, the simulation of physiological flow conditions during bacterial adhesion has significantly expanded the understanding of the pathogenesis of this type of infection (Pappelbaum et al., 2013; Liesenborghs et al., 2016; Claes et al., 2017). As for disseminated *S. aureus* infections, infections caused by *Escherichia coli* commonly develops at sites where considerable liquid shear stress needs to be overcome for the bacterium to successfully infect the host. By studying bacteria-host cell adhesion in a flow model, Thomas and colleagues found that the main *E. coli* adhesin, FimH, functions as a force sensor that increases adhesion strength to the uroepithelium when the bacterium is exposed to liquid shear (Thomas et al., 2002). Bacterial adhesion in flow models have also been used to show that *Borrelia burgdorferi* adheres to and detaches from the endothelium under flow in a fashion resembling leukocyte rolling on the vascular wall (Ebady et al., 2016), and that this attachment involves the recruitment of fibronectin thereby bridging the bacteria to the endothelial wall (Niddam et al., 2017). Furthermore, adhesion of *Neisseria meningitidis* to endothelial cells has been shown to depend on bacterial type IV pilus-induced remodeling of the endothelial plasma membrane enabling the bacterium to resist vascular shear forces (Mikaty et al., 2009; Soyer et al., 2014).

The above studies of the initial adhesion clearly demonstrate the importance of introducing physiological relevant fluid flow in studies of bacteria-epithelium interactions. Although initial adhesion is crucial, the ability of the bacterium to increase in numbers at the mucosal surface is the next challenge to overcome for successful establishment of infection. To simulate these events, prolonged infection experiments are needed and, especially when live human cell layers are employed, an environment must be created in which the human cell culture is viable despite the presence of much faster growing bacteria.

Only a few earlier studies have reported models that allow monitoring of simulated epithelial infection for prolonged periods of time (hours). Mairey et al. and Soyer et al. have demonstrated flow models used for studying *Neisseria meningitides* interaction with endothelial cell layers under flow over several hours. This model was used to investigate the adhesion to, colonization of and later detachment from these cell layers, and to perform studies of the influence of shear stress on the ability of the bacterium to interact with the capillaries of the brain (Mairey et al., 2006; Soyer and Duménil, 2011*)*. Alsharif et al. recently demonstrated a model for STEC intestinal infection in which the STEC strain O157:H7 Sakai was co-cultured on adherent HeLa cells under flow for up to 4 h, showing upregulation of central virulence genes as a result of host cell adhesion under liquid shear (Alsharif et al., 2015*)*. Using a microfluidic device, Kim et al. reported a co-culture model of HeLa cells and STEC that were used to demonstrate that the commensal biofilm microenvironment is a key determinant of STEC infectivity (Kim et al., 2010*)*. A recent model reported by Tremblay and co-workers also used a microfluidic device to investigate the ability of various pathogenic *E. coli* to colonize intestinal epithelial cell layers (Tremblay et al., 2015*)*. In the study, a protocol was demonstrated which supported initial STEC microcolony formation on the intestinal cell layer over 16 h and quantification of STEC growth based on analysis of fluorescent microscopy images of fixed infected cell layers after termination of the experiment.

Using a custom-built flow chamber setup, we previously demonstrated a urinary tract infection model featuring co-cultures of UPEC and uroepithelial cells, which allowed us to reproduce essential elements of *E. coli* uropathogenesis that have previously only been observed in animal models (Andersen et al., 2012; Khandige et al., 2016).

In the current study, we demonstrate an experimental methodology that enables prolonged infection assays and direct *in situ* quantification of the progression of bacterial colonization of epithelial cell layers under flow. Three infection models are presented, two of which use fixed uroepithelial or intestinal cell layers for simulation UPEC and STEC urinary tract and intestinal infection, respectively. The third model enables studies of adhesion and colonization of *S. aureus* on live endothelial cell layers under high-flow conditions, thereby simulating a key step in disseminated bloodstream infection. The methodology was specifically developed to allow accurate and sensitive estimation of colonization efficiency over time and for a better assessment of the importance of specific endogenous or exogenous factors in the ability of bacterial pathogens to colonize typical entrance sites in the human host.

While STEC is not *per se* considered a biofilm former, it does harbor a large number of specific genes that contribute to surface colonization and biofilm formation (Puttamreddy et al., 2010). In our study, we found that STEC strain EDL933 indeed is a solid biofilm former that effectively colonized the human intestinal epithelial layer during the 24 h experiment. Here, the use of fixed cell layers was required to prevent toxin-mediated killing of the cell layer, however, adhesion stimuli are likely to still be induced in the bacterium since fixed cells retain a significant amount of surface antigens (Yu et al., 2014). It should be emphasized though, that using fixed cell layers only provides a physiological substratum surface for bacterial growth, without further host cell-bacterium interaction, which is known to occur *in vivo* (Lai et al., 2013). If needed, studies focusing on these events, e.g., bacterial degradation of the epithelium, are possible using live epithelial cell layers instead and a suitable cell medium. This can for example be combined with the recording of fluorescence signals from virulence gene reporter-strains.

The model should be useful for elucidating the influence of specific virulence factors on intestinal colonization efficiency, as well as competition studies with commensal bacteria or more clinically orientated studies on the effect of antibiotic treatment. The latter is specifically important in the case of STEC intestinal infection, since antibiotic treatment has been reported to increase the risk of hemolytic uremic syndrome in patients with this type of infection (Freedman et al., 2016). Here, the model is a suitable *in vitro* tool for identification of more appropriate treatment regimens which to a lesser extent trigger the expression of virulence genes.

Endothelial colonization by *S. aureus* was conducted with live endothelial cells using of DMEM+FBS as flow medium. Due to the FBS content, this medium contains blood proteins that are used by *S. aureus* for adhesion, such as fibronectin. Of note, we have obtained preliminary results showing that the method can be conducted with live endothelial cells using diluted human plasma as only source of nutrients. This provides an even more physiological correct medium that both supports *S. aureus* growth and endothelial viability and further contains fibrinogen and coagulation components which are used by *S. aureus* to form thrombotic biofilms (Grønnemose et al., 2017).

The uroepithelial infection model presented here was conducted with the uroepithelial cell line HTB9 and the prototypical cystitis isolate UTI89. Based on morphology, HTB9 is a relatively end-stage differentiated cell line that provides a cell layer that strongly supports adhesion and invasion by UPEC (unpublished data). The drawback is that HTB9 is relatively fragile under high flow conditions and thus requires fixation to be used in the current assay. If live cells are needed to further study UPEC-uroepithelium interactions, the PD07i cell line (Klumpp et al., 2001) can be used in flow assays without fixation, as it is more flow resistant (Andersen et al., 2012) and stays confluent after prolonged infection experiments in urine flow (Klein et al., 2015). PD07i is however a relatively undifferentiated cell more resembling the underlying urothelial cells in the stratified bladder mucosa and, probably for this reason, is less supportive of UPEC adhesion/invasion (own unpublished data). To keep PD07i cells in their flow-resistant, undifferentiated growth mode it is maintained in specific and expensive serum-free, low calcium medium (Thumbikat et al., 2009) that adds significant costs to the experiments, in particular when high-flow conditions are used. It should also be mentioned that infection experiments with urine flow is incompatible with the current colonization quantification approach using fluorescence microscopy, since urine exhibits strong auto-fluorescence. The artificial urine (AU) used in the current study only exhibits very limited auto-fluorescence and, in contrast to urine specimens, does not vary in content and concentration. Although the AU is configured to closely simulate the chemistry of urine (Brooks and Keevil, 1997) and has the same effect as real urine on repressing T1F expression, we found in earlier studies that the AU does not have the same effect on UPEC growth when it comes to induction of bacterial morphology changes (Klein et al., 2015), which might influence surface colonization.

To demonstrate the application of the methodology, we investigated the influence of T1F on the capacity of UPEC to colonize uroepithelial cell layers. The importance of T1F in adhesion/invasion of uroepithelial cells is well-described from studies in static cell-culture assays (Martinez et al., 2000; Zhou et al., 2001; Martinez and Hultgren, 2002; Eto et al., 2007) and murine model studies (Connell et al., 1996; Bahrani-Mougeot et al., 2002). In the current study, we tested the influence of T1F on the entire progression from initial adhesion to biofilm establishment on an uroepithelial surface during a 24 h time period under a flow of AU. This experiment supported earlier studies in showing a significant influence of T1F on both adhesion and colonization of UPEC on the uroepithelial surface.

The role of T1F during UTI in humans has been associated with some discussion, since T1F is expressed to a limited extend *in vivo* (Lim et al., 1998; Hagan et al., 2010) and probably only among surface-associated sub-populations (Stærk et al., 2016). Experiments conducted in an earlier study to test if the lack of T1F on UPEC in the planktonic state in urine culture affected the ability of UPEC to form biofilm on catheter material, did not show a significant difference in the resulting 8 h biofilms (Stærk et al., 2016). When colonizing uroepithelium, however, UPEC binds to the uroepithelial cells via specific receptor-ligand adhesion through binding of the T1F adhesin FimH specifically to uroplakins on the superficial uroepithelial cells (Wu et al., 1996). Thus, the absence of T1F on UPEC growing in urine would be expected to considerably retard the bacterial capacity to bind and colonize the uroepithelium. In our assay, this was confirmed, showing a 6–10 h delay in surface colonization by the largely T1F-negative, wild-type UTI89 that were pre-cultured in AU. This delay corresponds well to the previously reported delay in transcriptional activation of the *fim* operon, which was first detected 8–12 h after initial adhesion (Stærk et al., 2016). The activation of the operon might result from a surface-sensing mechanism that transmits an intracellular signal, promoting the switching of the *fim*-switch promotor to the ON position. Alternatively, it might result from selective adhesion of a few T1F-positive UTI89 that lay the basis for a subsequent T1F-positive surface-population. Additional experimentation, e.g., with a *fim* GFP-reporter strain studied in the presented setup, should enable further elucidation of this mechanism.

In conclusion, the presented methodology renders possible detailed analysis of bacterial epithelium colonization capacity and opens up for uninterrupted investigation of initial host cell-pathogen interactions. A next step in the evolution of models to reproduce the interactions between the epithelium and bacteria is to control growth of differentiated epithelial cell layers, e.g., allowing cell exfoliation and 3D-cultures, as well as simulating the mucoid layers, which in some niches such as the intestine, is the first substance encountered by intestinal pathogens. Combined with the addition of immune cells and factors from the humoral immune response, it will be possible to simulate infection and inflammation responses with even greater precision. In addition to providing more detailed knowledge on host-pathogen interactions, this will enable scientists to better bridge *in vitro* experimental models with animal infection models.

## Author contributions

Study idea and design were conceived by RP, RG, and TEA. Experimental work was performed by RP, TBA, and CA. Manuscript writing was completed by RP, RG, KS, HK, JM-J, and TEA. All authors were involved in the discussion and interpretation of the results at all stages of the manuscript preparation.

### Conflict of interest statement

The authors declare that the research was conducted in the absence of any commercial or financial relationships that could be construed as a potential conflict of interest.

## References

[B1] Albert M. J. Grant T. Robins-Browne R. (2000). Studying bacterial adhesion to cultured cells, in Handbook of Bacterial Adhesion, eds An Y. H. Friedman R. J. (Totowa, NJ: Humana Press), 541–552.

[B2] Alsharif G. Ahmad S. Islam M. S. Shah R. Busby S. J. Krachler A. M. (2015). Host attachment and fluid shear are integrated into a mechanical signal regulating virulence in *Escherichia coli* O157:H7. Proc. Natl. Acad. Sci. U.S.A. 112, 5503–5508. 10.1073/pnas.1422986112 25870295 PMC4418854

[B3] Andersen T. E. Khandige S. Madelung M. Brewer J. Kolmos H. J. Møller-Jensen J. (2012). *Escherichia coli* uropathogenesis *in vitro*: invasion, cellular escape, and secondary infection analyzed in a human bladder cell infection model. Infect. Immun. 80, 1858–1867. 10.1128/IAI.06075-11 22354025 PMC3347433

[B4] Azeredo J. Azevedo N. F. Briandet R. Cerca N. Coenye T. Costa A. R.. (2017). Critical review on biofilm methods. Crit. Rev. Microbiol. 43, 313–351. 10.1080/1040841X.2016.1208146 27868469

[B5] Bahrani-Mougeot F. K. Buckles E. L. Lockatell C. V. Hebel J. R. Johnson D. E. Tang C. M.. (2002). Type 1 fimbriae and extracellular polysaccharides are preeminent uropathogenic *Escherichia coli* virulence determinants in the murine urinary tract. Mol. Microbiol. 45, 1079–1093. 10.1046/j.1365-2958.2002.03078.x 12180926

[B6] Berry R. E. Klumpp D. J. Schaeffer A. J. (2009). Urothelial cultures support intracellular bacterial community formation by uropathogenic *Escherichia coli*. Infect. Immun. 77, 2762–2772. 10.1128/IAI.00323-09 19451249 PMC2708588

[B7] Bouckaert J. Mackenzie J. de Paz J. L. Chipwaza B. Choudhury D. Zavialov A.. (2006). The affinity of the FimH fimbrial adhesin is receptor-driven and quasi-independent of *Escherichia coli* pathotypes. Mol. Microbiol. 61, 1556–1568. 10.1111/j.1365-2958.2006.05352.x 16930149 PMC1618777

[B8] Branda S. S. González-Pastor J. E. Ben-Yehuda S. Losick R. Kolter R. (2001). Fruiting body formation by *Bacillus subtilis*. Proc. Natl. Acad. Sci. U.S.A. 98, 11621–11626. 10.1073/pnas.191384198 11572999 PMC58779

[B9] Brock T. D. (1971). Microbial growth rates in nature. Bacteriol. Rev. 35, 39–58. 4929658 10.1128/br.35.1.39-58.1971 PMC378371

[B10] Brooks T. Keevil C. W. (1997). A simple artificial urine for the growth of urinary pathogens. Lett. Appl. Microbiol. 24, 203–206. 10.1046/j.1472-765X.1997.00378.x 9080700

[B11] Claes J. Liesenborghs L. Peetermans M. Veloso T. R. Missiakas D. Schneewind O.. (2017). Clumping factor A, von Willebrand factor-binding protein and von Willebrand factor anchor *Staphylococcus aureus* to the vessel wall. J. Thromb. Haemost. 15, 1009–1019. 10.1111/jth.13653 28182324 PMC6232194

[B12] Claes J. Vanassche T. Peetermans M. Liesenborghs L. Vandenbriele C. Vanhoorelbeke K.. (2014). Adhesion of *Staphylococcus aureus* to the vessel wall under flow is mediated by von Willebrand factor-binding protein. Blood. 124, 1669–1676. 10.1182/blood-2014-02-558890 24951431 PMC7025350

[B13] Connell I. Agace W. Klemm P. Schembri M. Mãrild S. Svanborg C. (1996). Type 1 fimbrial expression enhances *Escherichia coli* virulence for the urinary tract. Proc. Natl. Acad. Sci. U.S.A. 93, 9827–9832. 10.1073/pnas.93.18.9827 8790416 PMC38514

[B14] Djordjevic D. Wiedmann M. McLandsborough L. A. (2002). Microtiter plate assay for assessment of *Listeria monocytogenes* biofilm formation. Appl. Environ. Microbiol. 68, 2950–2958. 10.1128/AEM.68.6.2950-2958.2002 12039754 PMC123944

[B15] Ebady R. Niddam A. F. Boczula A. E. Kim Y. R. Gupta N. Tang T. T.. (2016). Biomechanics of *Borrelia burgdorferi* vascular interactions. Cell Rep. 16, 2593–2604. 10.1016/j.celrep.2016.08.013 27568563 PMC5235898

[B16] Edwards A. M. Potts J. R. Josefsson E. Massey R. C. (2010). *Staphylococcus aureus* host cell invasion and virulence in sepsis is facilitated by the multiple repeats within FnBPA. PLoS Pathog. 6:e1000964. 10.1371/journal.ppat.1000964 20585570 PMC2891841

[B17] Elbing K. Brent R. (2002). Media preparation and bacteriological tools. Curr. Protoc. Mol. Biol. Chapter 1:Unit 1.1. 10.1002/0471142727.mb0101s59 18265292

[B18] Eto D. S. Jones T. A. Sundsbak J. L. Mulvey M. A. (2007). Integrin-mediated host cell invasion by type 1-piliated uropathogenic *Escherichia coli*. PLoS Pathog. 3:e100. 10.1371/journal.ppat.0030100 17630833 PMC1914067

[B19] Finlay B. B. Falkow S. (1997). Common themes in microbial pathogenicity revisited. Microbiol. Mol. Biol. Rev. 61, 136–169. 9184008 10.1128/mmbr.61.2.136-169.1997 PMC232605

[B20] Freedman S. B. Xie J. Neufeld M. S. Hamilton W. L. Hartling L. Tarr P. I.. (2016). Shiga Toxin-Producing *Escherichia coli* infection, antibiotics, and risk of developing hemolytic uremic syndrome: a meta-analysis. Clin. Infect. Dis. 62, 1251–1258. 10.1093/cid/ciw099 26917812 PMC4845788

[B21] Greene S. E. Hibbing M. E. Janetka J. Chen S. L. Hultgren S. J. (2015). Human urine decreases function and expression of type 1 Pili in Uropathogenic *Escherichia coli*. MBio. 6:e00820. 10.1128/mBio.00820-15 26126855 PMC4488945

[B22] Grønnemose R. B. Saederup K. L. Kolmos H. J. Hansen S. W. K. Asferg C. A. Rasmussen K. J. (2017). A novel *in vitro* model for hematogenous spreading of *S*. aureus device biofilms demonstrating clumping dispersal as an advantageous dissemination mechanism. Cell. Microbiol. 19:e12785 10.1111/cmi.12785 28873268

[B23] Hagan E. C. Lloyd A. L. Rasko D. A. Faerber G. J. Mobley H. L. T. (2010). *Escherichia coli* global gene expression in urine from women with urinary tract infection. PLoS Pathog. 6:e1001187. 10.1371/journal.ppat.1001187 21085611 PMC2978726

[B24] Justice S. S. Hung C. Theriot J. A. Fletcher D. A. Anderson G. G. Footer M. J.. (2004). Differentiation and developmental pathways of uropathogenic *Escherichia coli* in urinary tract pathogenesis. Proc. Natl. Acad. Sci. U.S.A. 101, 1333–1338. 10.1073/pnas.0308125100 14739341 PMC337053

[B25] Justice S. S. Hunstad D. A. Seed P. C. Hultgren S. J. (2006). Filamentation by *Escherichia coli* subverts innate defenses during urinary tract infection. Proc. Natl. Acad. Sci. U.S.A. 103, 19884–19889. 10.1073/pnas.0606329104 17172451 PMC1750882

[B26] Khandige S. Asferg C. A. Rasmussen K. J. Larsen M. J. Overgaard M. Andersen T. E.. (2016). DamX controls reversible cell morphology switching in uropathogenic *Escherichia coli*. MBio 7, e00642–e00616. 10.1128/mBio.00642-16 27486187 PMC4981707

[B27] Kim J. Hegde M. Jayaraman A. (2010). Co-culture of epithelial cells and bacteria for investigating host-pathogen interactions. Lab Chip. 10, 43–50. 10.1039/B911367C 20024049

[B28] Klein K. Palarasah Y. Kolmos H. J. Møller-Jensen J. Andersen T. E. (2015). Quantification of filamentation by uropathogenic *Escherichia coli* during experimental bladder cell infection by using semi-automated image analysis. J. Microbiol. Methods. 109, 110–116. 10.1016/j.mimet.2014.12.017 25546841

[B29] Klumpp D. J. Weiser A. C. Sengupta S. Forrestal S. G. Batler R. A. Schaeffer A. J. (2001). Uropathogenic *Escherichia coli* potentiates type 1 pilus-induced apoptosis by suppressing NF-kappaB. Infect. Immun. 69, 6689–6695. 10.1128/IAI.69.11.6689-6695.2001 11598039 PMC100044

[B30] Ko Y. P. Kuipers A. Freitag C. M. Jongerius I. Medina E. van Rooijen W. J.. (2013). Phagocytosis escape by a *Staphylococcus aureus* protein that connects complement and coagulation proteins at the bacterial surface. PLoS Pathog. 9:e1003816. 10.1371/journal.ppat.1003816 24348255 PMC3861539

[B31] Krismer B. Liebeke M. Janek D. Nega M. Rautenberg M. Hornig G.. (2014). Nutrient limitation governs *Staphylococcus aureus* metabolism and niche adaptation in the human nose. PLoS Pathog. 10:e1003862. 10.1371/journal.ppat.1003862 24453967 PMC3894218

[B32] Kuthan M. Devaux F. Janderová B. Slaninová I. Jacq C. Palková Z. (2003). Domestication of wild *Saccharomyces cerevisiae* is accompanied by changes in gene expression and colony morphology. Mol. Microbiol. 47, 745–754. 10.1046/j.1365-2958.2003.03332.x 12535073

[B33] Lai Y. Rosenshine I. Leong J. M. Frankel G. (2013). Intimate host attachment: enteropathogenic and enterohaemorrhagic *Escherichia coli*. Cell. Microbiol. 15, 1796–1808. 10.1111/cmi.12179 23927593 PMC4036124

[B34] Lebeaux D. Chauhan A. Rendueles O. Beloin C. (2013). From *in vitro* to *in vivo* models of bacterial biofilm-related infections. Pathogens 2, 288–356. 10.3390/pathogens2020288 25437038 PMC4235718

[B35] Lembke C. Podbielski A. Hidalgo-Grass C. Jonas L. Hanski E. Kreikemeyer B. (2006). Characterization of biofilm formation by clinically relevant serotypes of group A streptococci. Appl. Environ. Microbiol. 72, 2864–2875. 10.1128/AEM.72.4.2864-2875.2006 16597993 PMC1449035

[B36] Letourneau J. Levesque C. Berthiaume F. Jacques M. Mourez M. (2011). *In Vitro* assay of bacterial adhesion onto mammalian epithelial cells. J. Vis. Exp. 2783. 10.3791/2783 21633326 PMC3197129

[B37] Liesenborghs L. Peetermans M. Claes J. Veloso T. R. Vandenbriele C. Criel M.. (2016). Shear-Resistant binding to von willebrand factor allows *Staphylococcus lugdunensis* to adhere to the cardiac valves and initiate Endocarditis. J. Infect. Dis. 213, 1148–1156. 10.1093/infdis/jiv773 26743845

[B38] Lim J. K. Gunther N. W. IV. Zhao H. Johnson D. E. Keay S. K. Mobley H. L. T. (1998). *In vivo* phase variation of *Escherichia coli* type 1 fimbrial genes in women with urinary tract infection. Infect. Immun. 66, 3303–3310. 9632599 10.1128/iai.66.7.3303-3310.1998 PMC108346

[B39] Loof T. G. Goldmann O. Naudin C. Mörgelin M. Neumann Y. Pils M. C.. (2015). *Staphylococcus aureus*-induced clotting of plasma is an immune evasion mechanism for persistence within the fibrin network. Microbiology 161(Pt 3), 621–627. 10.1099/mic.0.000019 25533444

[B40] Mairey E. Genovesio A. Donnadieu E. Bernard C. Jaubert F. Pinard E.. (2006). Cerebral microcirculation shear stress levels determine *Neisseria meningitidis* attachment sites along the blood-brain barrier. J. Exp. Med. 203, 1939–1950. 10.1084/jem.20060482 16864659 PMC2118386

[B41] Martinez J. J. Hultgren S. J. (2002). Requirement of Rho-family GTPases in the invasion of Type 1-piliated uropathogenic *Escherichia coli*. Cell. Microbiol. 4, 19–28. 10.1046/j.1462-5822.2002.00166.x 11856170

[B42] Martinez J. J. Mulvey M. A. Schilling J. D. Pinkner J. S. Hultgren S. J. (2000). Type 1 pilus-mediated bacterial invasion of bladder epithelial cells. EMBO J. 19, 2803–2812. 10.1093/emboj/19.12.2803 10856226 PMC203355

[B43] Mikaty G. Soyer M. Mairey E. Henry N. Dyer D. Forest K. T.. (2009). Extracellular bacterial pathogen induces host cell surface reorganization to resist shear stress. PLoS Pathog. 5:e1000314. 10.1371/journal.ppat.1000314 19247442 PMC2642725

[B44] Mulvey M. A. Schilling J. D. Hultgren S. J. (2001). Establishment of a Persistent *Escherichia coli* Reservoir during the Acute Phase of a Bladder Infection. Infect. Immun. 69, 4572–4579. 10.1128/IAI.69.7.4572-4579.2001. 11402001 PMC98534

[B45] Niddam A. F. Ebady R. Bansal A. Koehler A. Hinz B. Moriarty T. J. (2017). Plasma fibronectin stabilizes *Borrelia burgdorferi*-endothelial interactions under vascular shear stress by a catch-bond mechanism. Proc. Natl. Acad. Sci. U.S.A. 114, E3490–E3498. 10.1073/pnas.1615007114 28396443 PMC5410840

[B46] Novick R. P. (2003). Autoinduction and signal transduction in the regulation of staphylococcal 790 virulence. Mol. Microbiol. 48, 1429–1449. 10.1046/j.1365-2958.2003.03526.x 12791129

[B47] Palková Z. (2004). Multicellular microorganisms: laboratory versus nature. EMBO Rep. 5, 470–476. 10.1038/sj.embor.7400145 15184977 PMC1299056

[B48] Papaioannou T. G. Stefanadis C. (2005). Vascular wall shear stress: basic principles and methods. Hellenic J. Cardiol. 46, 9–15. Available online at: http://www.hellenicjcardiol.org/archive/full_text/2005/1/2005_1_9.pdf 15807389

[B49] Pappelbaum K. I. Gorzelanny C. Grässle S. Suckau J. Laschke M. W. Bischoff M.. (2013). Ultralarge von Willebrand factor fibers mediate luminal *Staphylococcus aureus* adhesion to an intact endothelial cell layer under shear stress. Circulation 128, 50–59. 10.1161/CIRCULATIONAHA.113.002008 23720451

[B50] Pizarro-Cerdá J. Cossart P. (2006). Bacterial adhesion and entry into host cells. Cell 124, 715–727. 10.1016/j.cell.2006.02.012 16497583

[B51] Proctor R. A. von Eiff C. Kahl B. C. Becker K. McNamara P. Herrmann M.. (2006). Small colony variants: a pathogenic form of bacteria that facilitates persistent and recurrent infections. Nat. Rev. Microbiol. 4, 295–305. 10.1038/nrmicro1384 16541137

[B52] Puttamreddy S. Cornick N. A. Minion F. C. (2010). Genome-wide transposon mutagenesis reveals a role for pO157 genes in biofilm development in *Escherichia coli* O157:H7 EDL933. Infect. Immun. 78, 2377–2384. 10.1128/IAI.00156-10 20351142 PMC2876562

[B53] Reisner A. Maierl M. Jörger M. Krause R. Berger D. Haid A.. (2014). Type 1 fimbriae contribute to catheter-associated urinary tract infections caused by *Escherichia coli*. J. Bacteriol. 196, 931–939. 10.1128/JB.00985-13 24336940 PMC3957706

[B54] Ribet D. Cossart P. (2015). How bacterial pathogens colonize their hosts and invade deeper tissues. Microbes Infect. 17, 173–183. 10.1016/j.micinf.2015.01.004 25637951

[B55] Riley L. W. Remis R. S. Helgerson S. D. McGee H. B. Wells J. G. Davis B. R.. (1983). Hemorrhagic colitis associated with a rare *Escherichia coli* serotype. N. Engl. J. Med. 308, 681–685. 10.1056/NEJM198303243081203 6338386

[B56] Roos V. Nielsen E. M. Klemm P. (2006). Asymptomatic bacteriuria *Escherichia coli* strains: adhesins, growth and competition. FEMS Microbiol. Lett. 262, 22–30. 10.1111/j.1574-6968.2006.00355.x 16907735

[B57] Rose C. Parker A. Jefferson B. Cartmell E. (2015). The characterization of feces and urine: a review of the literature to inform advanced treatment technology. Crit. Rev. Environ. Sci. Technol. 45, 1827–1879. 10.1080/10643389.2014.1000761 26246784 PMC4500995

[B58] Schnaith A. Kashkar H. Leggio S. A. Addicks K. Krönke M. Krut O. (2007). *Staphylococcus aureus* subvert autophagy for induction of caspase-independent host cell death. J. Biol. Chem. 282, 2695–2706. 10.1074/jbc.M609784200 17135247

[B59] Scholz O. Thiel A. Hillen W. Niederweis M. (2000). Quantitative analysis of gene expression with an improved green fluorescent protein. Eur. J. Biochem. 267, 1565–1570. 10.1046/j.1432-1327.2000.01170.x 10712585

[B60] Smith H. (1998). What happens to bacterial pathogens *in vivo*? Trends Microbiol. 6, 239–243. 10.1016/S0966-842X(98)01250-5 9675801

[B61] Sønderholm M. Kragh K. N. Koren K. Jakobsen T. H. Darch S. E. Alhede M.. (2017). *Pseudomonas aeruginosa* aggregate formation in an alginate bead model system exhibits *In Vivo*-like characteristics. Appl. Environ. Microbiol. 83, e00113–e00117. 10.1128/AEM.00113-17 28258141 PMC5394317

[B62] Soyer M. Charles-Orszag A. Lagache T. Machata S. Imhaus A. F. Dumont A.. (2014). Early sequence of events triggered by the interaction of *Neisseria meningitidis* with endothelial cells. Cell. Microbiol. 16, 878–895. 10.1111/cmi.12248 24320113

[B63] Soyer M. Duménil G. (2011). Introducing shear stress in the study of bacterial adhesion. J. Vis. Exp. e3241. 10.3791/3241 21912368 PMC3217250

[B64] Stærk K. Khandige S. Kolmos H. J. Møller-Jensen J. Andersen T. E. (2016). Uropathogenic *Escherichia coli* Express Type 1 fimbriae only in surface adherent populations under physiological growth conditions. J. Infect. Dis. 213, 386–394. 10.1093/infdis/jiv422 26290608

[B65] Thomas W. E. Trintchina E. Forero M. Vogel V. Sokurenko E. V. (2002). Bacterial adhesion to target cells enhanced by shear force. Cell 109, 913–923. 10.1016/S0092-8674(02)00796-1 12110187

[B66] Thumbikat P. Berry R. E. Schaeffer A. J. Klumpp D. J. (2009). Differentiation-induced uroplakin III expression promotes urothelial cell death in response to uropathogenic *E. coli*. Microbes Infect. 11, 57–65. 10.1016/j.micinf.2008.10.008 19007907 PMC2847841

[B67] Tremblay Y. D. Vogeleer P. Jacques M. Harel J. (2015). High-throughput microfluidic method to study biofilm formation and host-pathogen interactions in pathogenic *Escherichia coli*. Appl. Environ. Microbiol. 81, 2827–2840. 10.1128/AEM.04208-14 25681176 PMC4375333

[B68] Wright K. J. Seed P. C. Hultgren S. J. (2007). Development of intracellular bacterial communities of uropathogenic *Escherichia coli* depends on type 1 pili. Cell. Microbiol. 9, 2230–2241. 10.1111/j.1462-5822.2007.00952.x 17490405

[B69] Wu X. R. Sun T. T. Medina J. J. (1996). *In vitro* binding of type 1-fimbriated *Escherichia coli* to uroplakins Ia and Ib: relation to urinary tract infections. Proc. Natl. Acad. Sci. U.S.A. 93, 9630–9635. 10.1073/pnas.93.18.9630 8790381 PMC38479

[B70] Yu Y. Sun X. Guan X. Zhang X. Ma C. Chen L.. (2014). Effects of hydroformylation treatment on the storage time and blood group antigen expressions of reagent red blood cells. Transfus. Apher. Sci. 50, 462–466. 10.1016/j.transci.2014.02.019 24661843

[B71] Zapotoczna M. O'Neill E. O'Gara J. P. (2016). Untangling the diverse and redundant mechanisms of *Staphylococcus aureus* biofilm formation. PLoS Pathog. 12:e1005671. 10.1371/journal.ppat.1005671 27442433 PMC4956047

[B72] Zhou G. Mo W. J. Sebbel P. Min G. Neubert T. A. Glockshuber R.. (2001). Uroplakin Ia is the urothelial receptor for uropathogenic *Escherichia coli*: evidence from *in vitro* FimH binding. J. Cell. Sci. 114(Pt 22), 4095–103. Available online at: http://jcs.biologists.org/content/114/22/4095.article-info 11739641 10.1242/jcs.114.22.4095

