# Supplementary material for: A Method for Quantification of Epithelium Colonization Capacity by Pathogenic Bacteria
Source: Front Cell Infect Microbiol. 2018 Feb 1;8:16. doi: 10.3389/fcimb.2018.00016 (PMC5799267; doi:10.3389/fcimb.2018.00016)
Supplement: Supplementary file 1 [file DataSheet1.DOCX]

# Supplementary material

## Supplemental figure 1


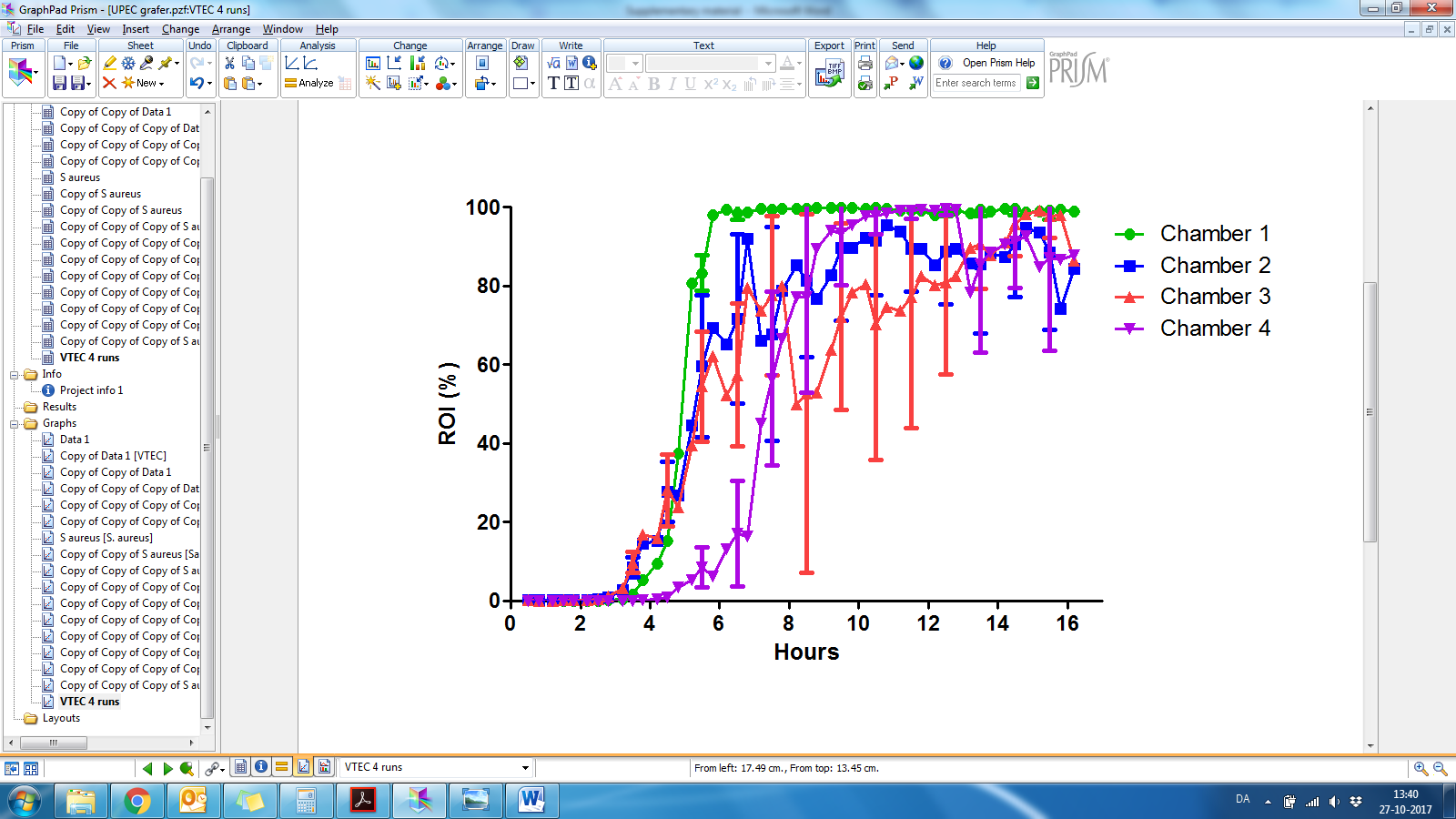


**Figure S1**. Shiga toxin-producing *Escherichia coli* strain EDL 933 colonization of fixed T84 cell layers. Graph illustrates biofilm coverage (detected GFP signal) as percentage of the monitored area (ROI-%). Values in each graph represent ROI-% at 11 randomly chosen 1100 µm x 850 µm sites in one flow chamber run. Data were recorded each 20 minutes throughout the experiment. Error bars indicate +/- 1 standard deviation between different positions in individual chambers. ROI, region of interest.

## Supplemental figure 2


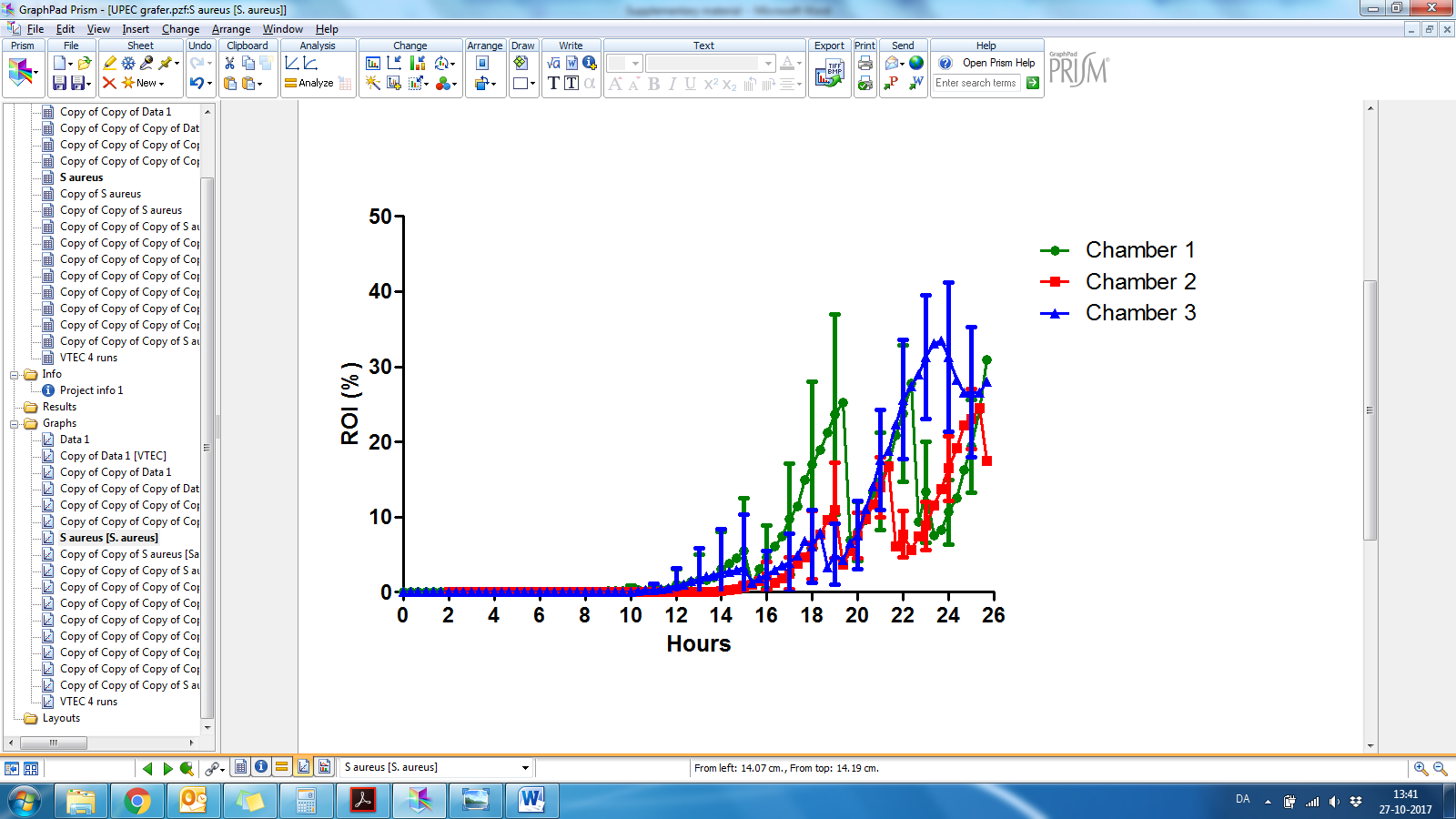


**Figure S2.** *Staphylococcus aureus* strain ATCC 29213 colonization of live EA.hy926 cell layers. Graph illustrates biofilm coverage (detected GFP signal) as percentage of the monitored area (ROI-%). Values in each graph represent ROI-% at 11 randomly chosen 1100 µm x 850 µm sites in one flow chamber run. Data were recorded each 20 minutes throughout the experiment. Error bars indicate +/- 1 standard deviation between different positions in individual chambers. ROI, region of interest.

## Supplemental figure 3


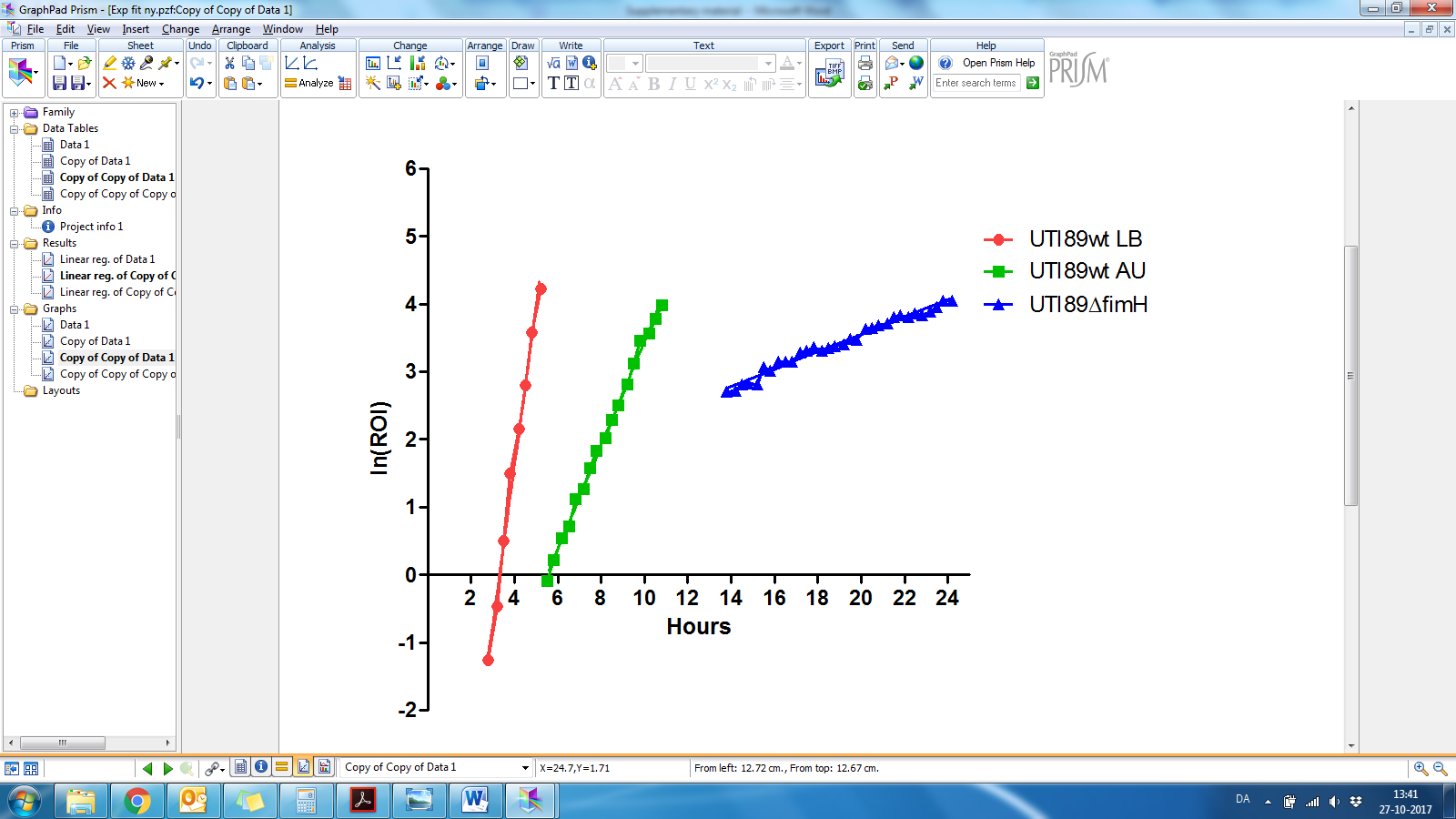


**Figure S3.** Data from experiments with UPEC strain UTI89 (Figure 5) transformed to ln and with fitted lines. Only exponential phase data are included.
